# L-gulono-γ-lactone Oxidase, the Key Enzyme for L-Ascorbic Acid Biosynthesis

**DOI:** 10.3390/cimb46100657

**Published:** 2024-10-01

**Authors:** Abdul Aziz M. Gad, Agnieszka Sirko

**Affiliations:** 1 Institute of Biochemistry and Biophysics, Polish Academy of Sciences, ul. Pawińskiego 5A, 02-106 Warsaw, Poland; asirko@ibb.waw.pl; 2 Molecular Biology Department, Biotechnology Research Institute, National Research Centre, Cairo 12622, Egypt

**Keywords:** ascorbic acid, L-gulono-γ-lactone oxidase, vitamin C, GULO characterization, C-terminal GULO, HWXK motif

## Abstract

L-ascorbic acid (AsA, vitamin C) plays a vital role in preventing various diseases, particularly scurvy. AsA is known for its antioxidant properties, which help protect against reactive oxygen species generated from metabolic activities; however, at high doses, it may exhibit pro-oxidative effects. The final step in AsA biosynthesis is catalyzed by L-gulono-γ-lactone oxidase (GULO). This enzyme is present in many organisms, but some animals, including humans, guinea pigs, bats, and other primates, are unable to synthesize AsA due to the absence of a functional *GULO* gene. The GULO enzyme belongs to the family of aldonolactone oxidoreductases (AlORs) and contains two conserved domains, an N-terminal FAD-binding region and a C-terminal HWXK motif capable of binding the flavin cofactor. In this review, we explore AsA production, the biosynthetic pathways of AsA, and the localization of GULO-like enzymes in both animal and plant cells. Additionally, we compare the amino acid sequences of AlORs across different species and summarize the findings related to their enzymatic activity. Interestingly, a recombinant C-terminal rat GULO (the cytoplasmic domain of the rat GULO expressed in *Escherichia coli*) demonstrated enzymatic activity. This suggests that the binding of the flavin cofactor to the HWXK motif at the C-terminus is sufficient for the formation of the enzyme’s active site. Another enzyme, GULLO7 from *Arabidopsis thaliana*, also lacks the N-terminal FAD-binding domain and is strongly expressed in mature pollen, although its activity has not been specifically measured.

## 1. Introduction

L-ascorbic acid (AsA), also known as vitamin C, is a naturally occurring organic compound belonging to the family of monosaccharides. Its consumption in humans correlates with increased protection against degenerative diseases and cancer [1]. The last stage of AsA production from glucose in mammals requires the enzyme L-gulono-γ-lactone oxidase (GULO), which is produced by the intermediates D-glucuronate and L-gulono-γ-lactone (GUL). Although numerous species are capable of synthesizing vitamin C, this enzymatic activity is lost in several mammals, such as humans, other primates, guinea pigs, some bat species, and insects [2]. The GULO enzyme belongs to the family of aldonolactone oxidoreductases (AlORs), which are a part of the vanillyl alcohol oxidase (VAO) family of flavoenzymes. When hydrogen peroxide is produced as a consequence of the oxidation of an aldonolactone using molecular oxygen as an electron acceptor, this family of enzymes is known as oxidases. If the reaction includes other electron acceptors, such as cytochrome c, phenazine methosulfate, 2,6-dichloroindophenol, or benzoquinone, these enzymes are referred to as dehydrogenases [3]. Plants appear to have multiple pathways for AsA biosynthesis. L-galactono-γ-lactone dehydrogenase (GalDH) catalyzes the conversion of GUL into AsA, while GULO-like enzymes, hereinafter referred to as GULLO, use L-galactono-γ-lactone (GAL) as a substrate [4].

## 2. The Role of Ascorbic Acid in Biochemical Processes and Its Biosynthetic Pathways

As an enzyme co-factor in hydroxylation reactions, AsA is vital to many processes in animals, including collagen synthesis [5], the demethylation of histones and nucleic acids [6], oxidative protein folding and endoplasmic reticulum (ER) stress [7], cell division and apoptosis [8], activation of the chloride channel regulator epithelial cystic fibrosis transmembrane conductance regulator [9], the production of surfactants in human lungs and immune homeostasis [10], macrophage function [11], and stress resistance. AsA has several uses as a medicinal product for human health, including the management of the common cold, wound healing, cancer, and scurvy prevention [5,12,13]. More recently, it has been found that diabetic complications due to uncontrolled hyperglycemia can be reduced if glucose is converted into AsA through GULO activation, thereby reducing oxidative stress [14]. The study by Kim et al. [15] states that endothelial cells can be stimulated to synthesize AsA intracellularly, by expressing GULO and providing them with the substrate GUL. Additionally, it was discovered that an increase in the amount of accessible tetrahydrobiopterin, which in turn enhanced NO generation in response to an increase in calcium, was caused by an increase in intracellular AsA. Therefore, by increasing NO bioactivity in endothelial cells, the intracellular administration of AsA may have positive effects on vascular physiology.

Grollman and Lehninger initially reported the absence of the GULO enzyme in mammals in 1957 [16], when they discovered that the vitamin C synthesis pathway is present in the kidney of birds and reptiles and the liver of mammals. In particular, they found that the three enzymes and the production of vitamin C were present in rats, mice, rabbits, dogs, cats, pigs, cows, chickens, pigeons, and turtles, but they were unable to detect the GULO enzyme in guinea pigs, rhesus monkeys, cynomolgus monkeys, or five distinct human species [5,16]. The four mammals without the third enzyme, according to Grollman and Lehninger, were required to be vitamin C consumers. However, a few years ago, clinical cases of scurvy in pigs were reported for the first time, thanks to the discovery of a mutant family of Danish pigs that is incapable of producing AsA [2,17]. 

There are other animal groups in which the deletion of the *GULO* gene has been reported, in addition to mammals. It is true that several species of Teleostei fish and Passeriformes birds do not carry the *GULO* gene. Despite the fact that vitamin C was found in numerous species [4,18,19], it was believed that the majority of protostomes lacked the *GULO* gene. There is little disagreement as to whether vitamin C is crucial for insects, despite the fact that they lack the *GULO* gene. For example, the L-ascorbate levels increase in *Drosophila melanogaster* Meigen, 1830 (Diptera: Drosophilidae), as they age, and vitamin C-enriched diets help flies live longer on average and to their maximum capacity [19,20]. The improved tolerance of the moth *Orgyia leucostigma* (J. E. Smith, 1797) (Lepidoptera: Erebidae) against tannin, a substance that induces reactive oxygen species (ROS), is thought to be caused by the increased amount of L-ascorbate found in its midgut lumen [21]. In response to several ROS-inducing pesticides, L-ascorbate levels decline in a dose-dependent way, potentially through supporting enzymatic antioxidant systems, including catalase, superoxide dismutase, and peroxidase [22]. It was discovered that the genes linked to L-ascorbate metabolism in the ladybird *Cryptolaemus montrouzieri* Mulsant, 1850 (Coleoptera: Coccinellidae) were selectively changed in reaction to cold stress, but not in response to heat stress [23]. *Caenorhabditis elegans* Maupas, 1900 (Rhabditida: Rhabditidae), a nematode, lacks the *GULO* gene; yet, it has been demonstrated that it can synthesize L-ascorbate from *Escherichia coli*, its food source. As a result, it has been suggested that *C. elegans* synthesizes L-ascorbate via a different enzyme or pathway, although the gene causing this has not been found [24]. 

Because most species lack a functioning *GULO* gene, they are unable to produce AsA. It is interesting to note that animal species lacking the *GULO* gene typically still have the other genes in the pathway, such as the *Regucalcin*/*SMP30* (Senescence Marker Protein 30) gene, which encodes for a gluconolactonase that generates the substrate of GULO (GUL) [4,25]. This finding implies that the other genes within the pathway have important roles. For instance, regucalcin functions as a transcription factor, a regulator of many nuclear processes, and a regulator of cellular Ca^2+^ levels in mammals [26,27]. Additionally, it has been noted that flies that grow without L-ascorbate exhibit elevated vitamin C levels in response to a brief 4 °C cold shock (10 min) [19,28]. 

The animals, including humans, that are unable to synthesize AsA must obtain this vitamin from commercially manufactured synthetic supplements or fresh fruits and green vegetables. The majority of AsA in the human diet comes from plants. In plants, AsA is the most prevalent and well-characterized water-soluble antioxidant [29]. AsA primarily builds up in the photosynthetic organs of plants, as it can be found in the cells of green tissues at concentrations of up to 5 mM, which is 10% of the total soluble carbohydrate pool [30]. In plant physiology, AsA is a critical metabolite that plays a key role in detoxifying ROS, promoting resistance to senescence, and mitigating the effects of a variety of environmental stressors, including high temperatures, dehydration stress, high light levels, ozone pollution, UV-B radiation, and salt stress. AsA also functions as a cofactor, contributing to the control of several essential cellular functions, such as photoprotection, the cell cycle, and cell division, as well as the production of important plant hormones, such as gibberellic acid, ethylene, abscisic acid, and jasmonic acid [31,32]. 

Prior to the characterization in *Arabidopsis thaliana* (L.) Heynh. (Capparales: Brassicaceae) of mutants (vtc-4-1 and vtc) that impair vitamin C production, the precise mechanism for vitamin C synthesis in plants was not known [30,33]. Currently, the Smirnoff–Wheeler pathway is regarded as the main mechanism used in the synthesis of vitamin C. While some plant species, like *Citrus* sp. and the kiwifruit *Actinidia chinensis* Planch. (Ericales: Actinidiaceae), are well-known for having high vitamin C content [34], the biosynthesis of vitamin C in *A. thaliana* has a well-established mechanism. The enzymes responsible for vitamin C biosynthesis in *A*. *thaliana* from glucose are hexokinase 1 (At4g29130, HXK1), phosphoglucose isomerase (At4g24620, PGI), phosphomannose isomerase 2 (At1g67070, DIN9, PMI2), phosphomannomutase (At2g45790, PMM), GDP-mannose pyrophosphorylase 1 (At2g39770, VTC1, GMP1), GDP-d-mannose epimerase (At5g28840, GME), GDP-L-galactose phosphorylase (At4g26850, VTC2, GGP), L-galactose 1-phosphate phosphatase (At3g02870, VTC4, GPP), L-galactose dehydrogenase (At4g33670, GalDH), and L-galactono-γ-lactone dehydrogenase (At3g47930, GLDH). The roles of these enzymes and their homologues in plants, and in the yeasts, *Kluyveromyces lactis* and *Saccharomyces cerevisiae,* have been established by research [35,36,37].

**Figure 1:**
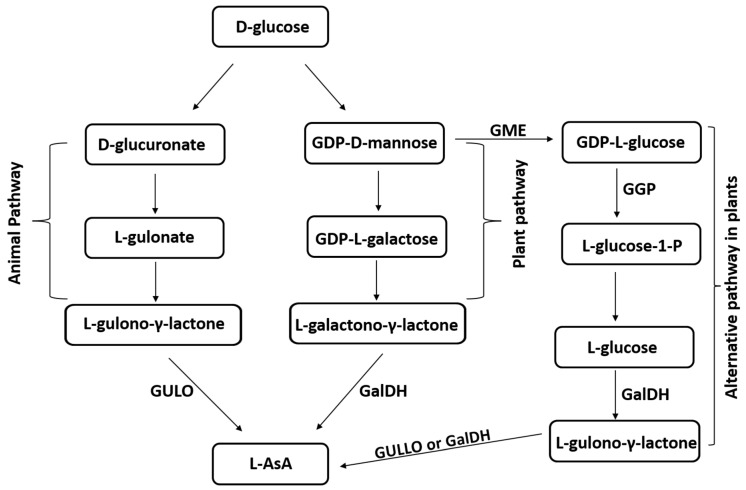
Main pathways for L-ascorbic acid (L-AsA) biosynthesis in animals and plants. GULO, animal L-gulono-γ-lactone oxidase; GULLO, plant L-gulono-γ-lactone oxidase; GalDH, L-galactono-γ-lactone dehydrogenase; GME, GDP-d-mannose epimerase; GGP, GDP-l-galactose phosphorylase [43,44,45].

Plants and animals have different AsA biosynthesis routes (Figure 1). The primary and most well-understood mechanism in plants is the “Wheeler–Smirnoff pathway”, sometimes referred to as the “D-mannose/L-galactose pathway” or the “plant pathway.” AsA biosynthesis from glucose or mannose is the first step [4,38], during which D-mannose is converted into GDP-D-mannose and then into GDP-L-galactose. AsA is a precursor to L-galactose, which is further oxidized to form L-galactono-γ-lactone [38]. Every gene involved in this process has been discovered and, at the end of the pathway, GalDH catalyzes an enzyme reaction that forms AsA from GAL. Plants appear to have alternative pathways for the biosynthesis of AsA that involve galacturonate and glucuronate; however, not all of the enzymes involved in these processes have been identified, and the regulation of these pathways is still unclear [39,40]. Moreover, it appears that a portion of the animal system, often referred to as the myo-inositol pathway, which uses GUL as the last precursor for AsA biosynthesis, is active in plants, albeit the specific enzymes involved have not yet been found [41,42]. GalDH serves as the terminal enzyme in the primary plant AsA biosynthesis pathway, whereas GULO serves this function in animals [4,13]. However, plants possess GULO-like enzymes (e.g., GULLO1-GULLO7 in *A. thaliana*), which may be involved in AsA formation via the alternative pathway involving GUL, as shown in Figure 1. The functionality of this pathway is suggested by the elevation in AsA production, due to the overexpression of rat GULO or *A. thaliana* GULLO5 in plants [43,44,45]. 

## 3. Methods of Ascorbic Acid Production

### 3.1. Manufacturing for Commercial Purposes

Among the 13 vitamins, AsA is the one that has the greatest industrial production volume. Every year, the pharmaceutical industry uses about 110 kilotons of vitamin C, which is manufactured industrially. Food and beverage companies use 25% and 15% of these kilotons of vitamin C as antioxidants, respectively. Animal feed applications only consume roughly 10% of the vitamin. This is not the case for any other vitamin, where the primary application for other vitamins is in the feed industry [46]. Chinese manufacturers supply more than 80% of the vitamin C needed by the global market today. The Reichstein process, which combines chemical and microbiological methods, is commonly used to generate commercial vitamin C from D-glucose [47]. Despite being in use for more than 60 years, the process has several inherent drawbacks, including lengthy production time, intricate procedures, high temperature and pressure requirements, and challenges associated with continuous operation. Furthermore, the Reichstein method necessitates the use of numerous hazardous chemicals that are energy intensive and have the potential to significantly contaminate the environment. As a result, scientists have looked for fresh approaches to enhance the effectiveness of the conventional Reichstein technique [48,49]. For instance, a two-step fermentation technique was developed that is easier to use and more ecologically friendly than the Reichstein process [50]. Unfortunately, the two-step fermentation method requires three separate strains of bacteria, which makes it difficult to convert D-glucose directly into vitamin C. A lot of work has gone into creating a one-step fermentation technique for vitamin C [51]. This has, in theory, made it possible to produce vitamin C directly from D-glucose; nevertheless, practically all the existing one-step vitamin C fermentation procedures generate 2-keto-L-gulonic acid (2-KLG) rather than vitamin C [52,53,54]. Additionally, there has been report about the direct production of vitamin C from D-glucose by engineering the ten genes involved in the vitamin C biosynthetic pathway in *A. thaliana* [37]. 

### 3.2. Recombinant Vanillyl Alcohol Oxidase Family Members Are Usually Produced in Bacterial Cells in Apo Forms Lacking the Flavin Cofactor

GULO was created in both holo and apo forms in riboflavin-rich and free media in insect cells, utilizing the baculovirus system. L-flavin adenine dinucleotide (FAD) only bound non-covalently to the apo form when added externally [55]. However, the enzymatic activity was the same as the holo form when FAD was covalently bound. In contrast, *E. coli* was shown to express protozoan GULO isoenzymes from *Trypanosoma brucei* Dutton, 1902 [56] and *Trypanosoma cruzi* Chagas, 1909 (Trypanosomatida: Trypanosomatidae) [57,58], as well as GalDH from *A. thaliana* [59] and tobacco [60], all of which possess a non-covalent flavin. In more detail, boiling or treating the protein with acid can liberate the redox active flavin cofactor, verifying the non-covalent binding mechanism that was previously inferred from the amino acid sequences. Remarkably, a recombinant *T. cruzi* L-galactono-γ-lactone oxidase (GALO) was efficiently refolded in a reverse micellar system into a native-like structure in the presence of FAD, but not with the flavin mononucleotide [58], which was localized in the inclusion bodies of *E. coli* following overexpression. When expressed in *E. coli*, GALO in *Leishmania donovani* Ross, 1903 (Trypanosomatida: Trypanosomatidae), which was predicted to have non-covalently bound FAD, was determined to be in the apo form, because no activity was seen without the addition of FAD [61]. An enzyme with covalently attached FAD was not produced by AlORS expression in *E. coli*. Given that several members of the vanillyl alcohol oxidase (VAO) family, including VAO itself [62], eugenol oxidase [63], alditol oxidase [64], and chitooligosaccharide oxidase [65], were successfully expressed as holo proteins in *E. coli*, it is possible that the recombinant proteins did not fold correctly. The non-flavoprotein may have been produced by the improper folding of recombinant *Mycobacterium tuberculosis* L-gulono-γ-lactone dehydrogenase (GLDH), expressed in *E. coli*.

### 3.3. Genetic Engineering of Plants and Animals to Elevate the Ascorbic Acid Level

It has been more than three decades since the idea of employing plants as production machines for different proteins, with biotechnological or medicinal value, was first proposed. Since 1989, biofactory plants have been producing the previously mentioned recombinant human protein. Using mammalian expression models, recombinant protein product yields are poor, and production costs are high [66]. Bacteria have long been utilized as an expression system for the synthesis of recombinant proteins, because of their straightforward molecular biology, the low cost of manufacturing them, and the ease of handling. The absence of post-translational machinery and the creation of inactive proteins as a result of improper disulfide bond formation, which can cause protein misfolding and aggregation into inclusion bodies, represent the biggest obstacles [67]. Instead, plants can produce fully folded functional molecules with nearly identical properties to the native protein and carry out all of the post-translational modifications required for proteins to have the best biological activity, thus plant cells are more attractive for producing eukaryotic proteins [68,69]. Transgenic plants can effectively manufacture recombinant proteins, such as antibodies, cytokines, and vaccines [70,71]. The most often utilized transgenic plants include tobacco, potato, banana, and *A. thaliana* [72].

The expression of even a single gene from the animal pathway can result in a significant increase in the ascorbic acid level in plants. Transgenic plants that express rat cDNA encoding GULO, such tobacco, potatoes, tomatoes, and *A. thaliana*, produce basal levels of ascorbic acid that can be up to seven times higher than those in untransformed plants [73,74,75]. In an effort to elevate the AsA level, a number of ascorbate biosynthesis pathway genes have recently been inserted into plants using metabolic engineering. These include a human *dehydroascorbate reductase* gene inserted into tobacco [76], GULO cDNA isolated from mice and inserted into tobacco and lettuce [73], and D-galacturonic acid reductase cDNA from strawberry inserted into *A. thaliana* and potato [39,77], and wheat dehydroascorbate cDNA inserted into tobacco and maize [78]. Recombinant GULLO5 (At2g46740) from *A. thaliana* was also transiently expressed in *Nicotiana benthamiana* Domin (Solanales: Solanaceae) and its activity was verified through ascorbic acid production [38]. In this context, three *Arabidopsis* GULO-like enzymes (GULLO2, 3, 5) overexpressed in tobacco BY-2 cell cultures increased the AsA level after being fed with L-GUL. The GULLO proteins from *A. thaliana* exhibit some similarity to animal GULO [79]. The production of a FAD-containing protein by GULLO-expressing vector-infected cells, matched in size to the expected recombinant GULLO protein, and the conversion of GUL to AsA, indicated the existence of a functional enzyme in cell-free extracts [80]. Also, in plant or animal cells, the expression of the rat or mouse *GULO* gene has been used to restore vitamin C biosynthetic capacity. The examples include the teleost fish, rainbow trout, and guinea pig cells [81], human cells [80], monkey cells [80,82], and the Far Eastern catfish [83,84]. 

## 4. Localization and Enzymatic Properties of GULO-like Enzymes from Different Species

It is interesting to note that as a result of evolution, the location of GULO expression appears to have changed from the kidney to the liver; whereas primitive birds, reptiles, and amphibia create GULO in the kidney, mammals and highly evolved birds manufacture it in the liver [2,17]. The GULOs in animals, such as rats [85], goats [3], mice [86], chickens [87], and pigs [2], were localized to microsomes and they are all integral membrane proteins. The rat GULO amino acid sequence has multiple potential membrane-associating sites [88]. The rat GULO was revealed to have an intraluminal active site, and it discharges its products into the ER lumen [89]. Although the exact location of the GULO active site is unknown, it is believed that the enzyme produces H_2_O_2_ and ascorbate on the lumenal side of the ER membrane [90,91]. 

GalDH, the final enzyme in the primary AsA biosynthesis pathway in plants, is connected to respiratory Complex 1 and is found in the inner membrane of mitochondria. GalDH may be connected to the respiratory electron transport chain in the mitochondria, based on its location and Cyt c selectivity. Even though a specific ascorbate transporter may be needed for transportation across the inner mitochondrial membrane, which is impermeable to sucrose, but selectively permeable to a limited number of anions depending on the orientation of GalDH, GAL may diffuse through the outer mitochondrial membrane, which is highly permeable to solutes like sucrose, nucleotides, and NAD. GalDH is expected to traverse the inner mitochondrial membrane based on its predicted location, but since its catalytic site faces the intermembrane gap, transporters would not be necessary [45,92,93,94]. Moreover, in *A. thaliana,* the GULO-like enzyme, GULLO5, localizes to the cell wall [95]. A similar localization to the cell wall occurs in regard to *M. tuberculosis* GalDH [96]. In *A. thaliana,* GalDH, a gatekeeper amino acid residue, was shown to be responsible for determining whether the oxidoreductase was an oxidase or a dehydrogenase. It was discovered that, with a few exceptions, alanine can be found in dehydrogenases, while glycine or proline can be found in oxidases [97]. Despite containing proline and glycine, respectively, *A. thaliana* GULLO5 and *M. tuberculosis* GLDH do not exhibit oxidase activity. It is important to note that the recombinant *M. tuberculosis* GLDH lacks a flavin, which may have contributed to its lack of reactivity with molecular oxygen [84,98]. 

Trypanosomal D-arabinono-γ-lactone oxidase (ALO) enzymes are found within glycosomes and can be specifically targeted to glycosomes due to a distinctive SHL motif at the C-terminus of their amino acid sequence [56,57]. Along with cytoplasm, *L. donovani* ALO is also found in glycosomes. The SHL motif is absent from the amino acid sequence [43,61].

In contrast, the subcellular localization of a GULLO from the mushroom *Grifola frondosa* (Dicks.) Gray (1821) (Polyporales: Meripilaceae) was not determined, but it may be a membrane protein [99]. This enzyme used GUL as the substrate and oxygen as the electron acceptor under aerobic circumstances to create AsA and H_2_O_2_. Anaerobic conditions led to the production of AsA, using GUL as the substrate and phenazine methosulfate as the electron acceptor. This means that in addition to being an oxidase, this enzyme also possesses dehydrogenase properties. Even though GULLO from *G. frondosa* has a high substrate specificity comparable to that of GalDHs in plants, its real substrate might be unknown [99,100].

**Figure 2:**
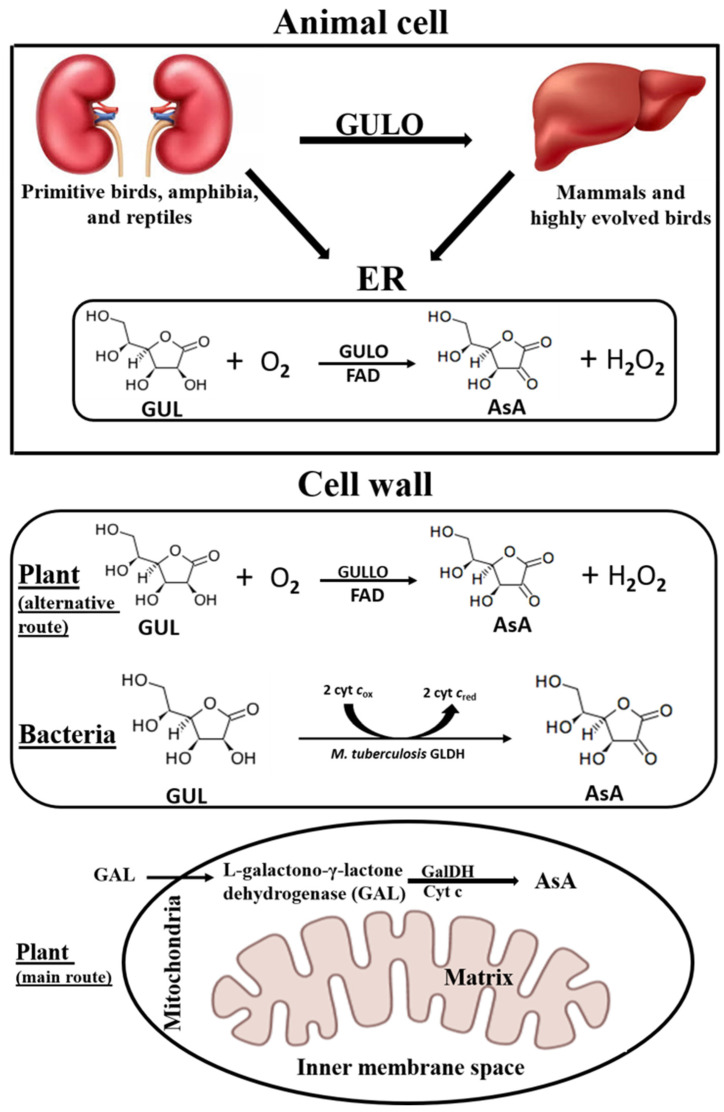
A comparison between the sites for ascorbic acid synthesis in animal, plant, and bacterial cells. AsA, L-ascorbic acid; ER, endoplasmic reticulum; GUL, L-gulono-γ-lactone; GULO, animal L-gulono-γ-lactone oxidase; GULLO, plant L-gulono-γ-lactone oxidase; GLDH, L-gulono-γ-lactone dehydrogenase; GAL, L-galactono-γ-lactone; GalDH, L-galactono-γ-lactone dehydrogenase; Cyt c, cytochrome c.

In summary, mammalian GULOs containing a covalently bound 8α-N1-histidyl FAD [101,102] are localized in microsomes, with the active site facing the lumen of the ER [89], protozoan isozymes are localized in glycosomes, while the localization of mushroom isoforms is unknown. In plants, the final step in AsA synthesis via the main pathway (GalDH) is localized in mitochondria [103], while the GULO-like enzymes, presumably responsible for the minor pathway, might be located near the cell wall (Figure 2).

## 5. Characterization of L-gulono-γ-lactone Oxidases from Different Sources

The GULO enzyme belongs to the AlORs family and contains two conserved domains: a C-terminal HWXK motif that can bind the flavin cofactor and an N-terminal FAD-binding region. All AlORs found in animals, fungi, and yeast are oxidases, but they also have dehydrogenase-like activity and use phenazine methosulfate as an electron acceptor rather than cytochrome c [104]. Protozoan enzymes consume oxygen and cytochrome c, and they are also oxidases and dehydrogenases [43]. Utilizing phenazine methosulfate and cytochrome c, plant GalDHs are exclusive dehydrogenases. All plant GalDHs contain the same amino acid, which was found to be the cause of oxygen reactivity in *A. thaliana* GalDH [43]. Exclusive dehydrogenases are also seen in bacterial enzymes. An isoenzyme from algae is special in that it can only take electrons in vitro from phenazine methosulfate and not from oxygen or cytochrome c [43]. Animal GULOs are microsomal enzymes that catalyze the interaction by GUL, with oxygen acting as an electron acceptor, to create hydrogen peroxide and L-ascorbate [84,91]. They use FAD as a cofactor in this reaction. It is commonly known that the flavin moiety has a role in the enzymatic catalysis of many AlORs. Reactions containing flavin proceed in two phases. The substrate reduces flavin and the latter is oxidized during the first half of the reductive process. Electron acceptors, like oxygen (oxidase), or other electron acceptors (dehydrogenases), reoxidize flavin in the second half of the process. Electrons from flavin are transferred to oxygen, producing hydrogen peroxide. Because of the distinctive spectrum characteristics displayed throughout the oxidation and reduction cycles involving the flavin group, both half reactions can be investigated independently [43,97]. GUL can be converted into AsA by all known GalDHs from plants [42] and from the protist *Euglena* sp. [104], which use cytochrome c and phenazine methosulfate, respectively, as an electron acceptor. Another example is the *M. tuberculosis* GLDH, which has the ability to receive exogenous electrons from both GUL and cytochrome c [105]. Plant GalDH and animal GULO are both active in terms of the GAL substrate. In the same situation, the *A. thaliana* GalDH uses cytochrome c as an electron acceptor to oxidize GUL, in addition to GAL [59]. In recent studies, Sivadas et al. [14] reported that some phytomolecules, as flavonoids with antioxidant properties, are hypothesized to be promising and selective activators of GULO. In contrast, the majority of the identified AlORs were inhibited by metal ions; Hg^+2^, Zn^+2^, and Cu^+2^ were discovered to be prevalent inhibitors of AlORs and were linked to inhibition through sulfhydryl groups [61,106]. By blocking flavin reduction, Hg^+2^ suppresses rat GULO activity, as shown by the absence of a flavin-corresponding peak at 450 nm. On the other hand, sulphite inhibited oxidase activity, but did not inhibit flavin reduction. Although the thiol group is only involved in substrate binding and not in catalysis, it is still essential for the function of these enzymes. This function involves just one sulfhydryl group, which was found to be Cys-340 in the GLDH in *A. thaliana* [43]. The existence of two active sites, one for oxidase activity and the other for dehydrogenase activity was, therefore, postulated by the scientists [84,107]. Reactions involving phenazine methosulfate may also feature a third active site that is independent of flavin, in addition to these two active sites [106]. Similarly, GLDH activity was higher with phenazine methosulfate and lower with cytochrome c, even though *M. tuberculosis* lacked a flavin prosthetic group [84,105]. 

Burns et al. [108] discovered GULO activity in rat liver microsomes for the first time. The enzyme from rat liver microsomes was partially purified by Eliceiri et al. [85]. Later, using the microsomes from rat and goat liver [3], as well as chicken kidney [87], GULO was isolated to apparent homogeneity and biochemically characterized (Table 1). This helped generate antisera, which in turn helped identify the cDNA sequence [88]. With the use of these instruments, the molecular process behind the deficiency of vitamin C production in scurvy-prone animals, including humans, was studied [109]. Recombinant proteins for both the full-length rat GULO (fGULO) and its C-terminal catalytic domain (cGULO) have recently been purified and biochemically characterized by Gad et al. [110]. A comparison of the biochemical properties of some previously purified and characterized members of the ALO family is shown in Table 1. 

As already mentioned above, GalDH and GULLO are both involved in AsA biosynthesis in plants. The GalDH activity in plants was first identified in the mitochondria of pea and mung bean seeds [111]. The enzyme responsible for this activity was partially purified and characterized from cauliflower floret mitochondria [107]. Since then, isoforms from spinach [112] and white potato tubers [113] have been reported, and cDNA sequences encoding GalDH from cauliflower [114] and sweet potato [115] were identified. The GULLO activity has also been detected in kidney bean hypocotyl homogenates [116], *Grifola frondosa* fruiting bodies [99], *A. thaliana* cell suspension of cytosolic and mitochondrial fractions [41]. The GULLO-like protein from the velvet bean *Mucuna sempervirens* Hemsl. (Fabales: Fabaceae) (homolog of GULO-like enzymes from *A. thaliana*) was purified to near homogeneity from *M. sempervirens* floral nectar and its enzymatic activity was assayed in vitro [13]. Additionally, Maruta et al. [79] expressed four of the seven putative GULLOs from *A. thaliana* in BY2 cell suspension cultures. A recombinant *A. thaliana GULLO5* was expressed in a *N. benthamiana*-based transient expression system and successfully characterized by Aboobucker et al. [43]. 

## 6. GULO-like Enzymes from *A. thaliana* and GULO from Rat

**Figure 3:**
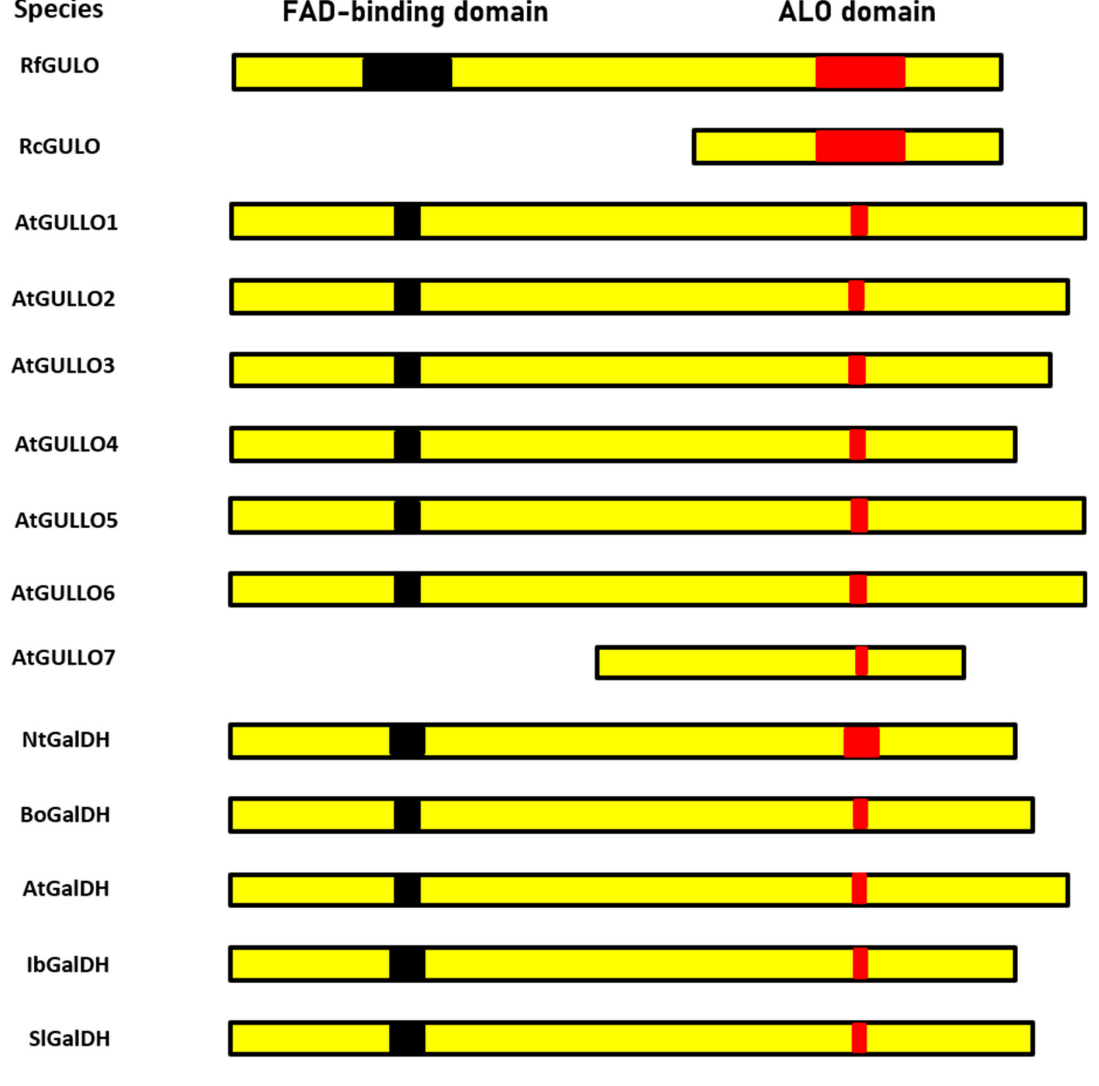
Schematic representation of the FAD-binding and ALO domains found in full-length rat GULO (RfGULO), C-terminal rat L-gulono-γ-lactone oxidase (RcGULO), *Arabidopsis* GULLOs and L-galactono-γ lactone dehydrogenase (GalDH) from different plant sources. Domain sequences of individual proteins were obtained from conserved domains in the NCBI Protein database and compared to that of rat fGULO using the Global Align program. The length of the domains was drawn in relation to RfGULO, which is assumed to be 100%. The accession numbers of the sequences used are as follows: RfGULO (P10867.3), *Arabidopsis* homologs (AtGULLO1 to AtGULLO7), and GalDHs from *Nicotiana tabacum* (NtGalDH; BAB13368.1), *Brassica oleracea* (BoGalDH; O47881.1), *Arabidopsis thaliana* (AtGalDH; NP_190376.1), *Ipomoea batatas* (IbGalDH; BAA34995.1), and *Solanum lycopersicum* (SlGalDH; NP_001234603.1) [43,110].

Seven GULO-like proteins identified in *A. thaliana* were named as GULLO1 to GULLO7 [79]. A schematic comparison of several representative *A. thaliana* ALO family members, recombinant rat GULO proteins, and GalDH from several plants, is shown in Figure 3. Most of the compared enzymes contain two conserved domains: the FAD-binding domain (Pfam Id: 01565) at the N-terminus and an ALO catalytic domain (Pfam Id: 04030) at the C-terminus [84,108].

Additionally, the domain sequences of *A. thaliana* GULLO1-7 were individually compared to those of the rat full-length L-gulono-γ-lactone oxidase (RfGULO), using BLAST2seq to generate a similarity score. The similarity of the ALO domains is 20–33% when compared to rat GULO and that of the FAD-binding domains is 28–36% (with the exception of GULLO7 which is N-terminally truncated). It might be speculated that GULLO7, which lacks the FAD-binding domain, may need a hypothetical effector molecule that is produced under stress conditions or in specific plant parts (mature pollen) where it is presumably present. Different mechanisms of post-translational regulation were discussed in terms of GULLO3 and GULLO5. For example, an effector molecule may increase the catalytic efficiency of GULLO5, while GULLO3 may use another mechanism to maintain its stability in regard to proteolytic degradation [43]. The speculation about the possible catalytic activity of GULLO7 is supported by the fact that the recombinant cytoplasmic domain of rat GULO (cGULO) expressed in *E. coli* is catalytically active, although it lacks the FAD-binding domain [110]. Presumably, only the flavin cofactor, linked to a recently discovered HWXK motif at the C-terminus, is crucial for the formation of the active site of this enzyme [43,110]. However, the enzymatic activity of *A. thaliana* GULLO7 remains to be verified.

## 7. The Family of Aldonolactone Oxidoreductases with the Conserved HWXK Motif

Specific to the ALO domain, engaged in the last stage of AsA biosynthesis, is the C-terminal HWXK motif. Plants and protozoa contain non-covalently bound FAD, while animals and fungi contain covalently bound FAD. An N-terminal histidine residue in flavoprotein AlORs facilitates the covalent interaction between the FAD cofactor and the C-terminal histidine residue [57,117]. The covalent connection inhibits flavin modification and boosts the enzyme redox power, cofactor saturation, and protein stability [118]. The cofactor shields the enzyme against irreversible unfolding or aggregation, as evidenced by the significant increase in thermal stability observed in the presence of excess FAD [118]. A mutation in the FAD-binding region may be a factor in the activity decrease observed in *Pteropus vampyrus* Linnaeus 1758 (Chiroptera: Pteropodidae) [119]. For this reason, ALO activity could not be detected in *L. donovani* in the absence of FAD [61]. The VAO family includes AlORs, and FAD attaches to a histidine in the C-terminus of VAO. Fraaije et al. [120] found that FAD is covalently linked to the His-422 residue in the C-terminus of VAOs. A histidine is found in all covalently bound FAD-containing isoenzymes, whereas a leucine or lysine is found in non-covalent FAD-containing enzymes. 

**Figure 4:**
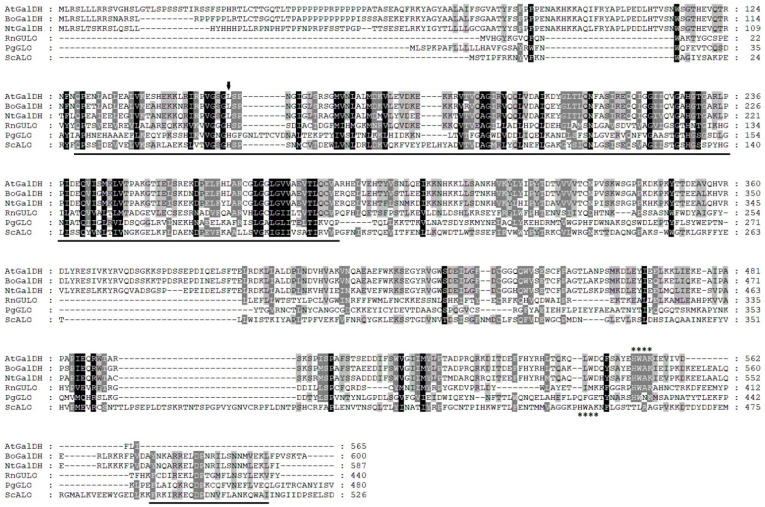
Multiple sequences of the full-length amino acid sequence of *A. thaliana* AtGalDH (At3g47930), with several aldonolactone oxidoreductases. The accession numbers (NCBI Entrez Protein Database) used for the multiple sequence alignment are *Brassica oleracea* BOGalDH (CAB09796), *Nicotiana tabacum* NtGalDH (BAA80870), *Rattus norvegicus* RnGULO (P10867), *Penicillium griseoroseum* gluconolactone oxidase PgGLO (AAT80870), and *Saccharomyces cerevisiae* ScALO (P54783). Alignment was performed using CLUSTAL X2. Amino acid residue numbers are shown on the right. Identical residues are shaded in black; similar residues are shaded in gray. The arrow marks the histidine residue involved in covalent binding of the FAD cofactor in GUO, ALO, and GLO, or the leucine residue in GalDH proteins. The FAD-binding domain in both the N-terminal and C-terminal parts is underlined. The asterisks mark the HWAK motif in RnGULO, ScALO, and all GalDH proteins.

To highlight the most conserved regions of the ALO family, we performed a multiple sequence alignment of several representative members from different organisms: *A. thaliana* AtGalDH, *Brassica oleracea* BoGalDH, *Nicotiana tabacum* NtGalDH, *Rattus norvegicus* RnGULO, *Penicillium griseoroseum* gluconolactone oxidase PgGLO, and *Saccharomyces cerevisiae* ScALO (Figure 4). The FAD-binding domain of the enzymes exhibited the highest level of sequence conservation. The alignment makes it evident that plant GalDHs contain a leucine residue (indicated by the arrow), rather than the histidine residue required for covalent flavinylation in GULO, ALO, and GLO. This suggests that the flavin cofactor is non-covalently attached to the protein. Noticeably, the histidine in the HWXK motif located in the C-termini of all AlORs is evolutionarily conserved [43,57]. The functional significance of the HWXK motif in the *T. cruzi* GALO was investigated through the use of site-directed mutagenesis [57]. Individual mutations of His-447 and Trp-448 to glycine resulted in the loss of flavin binding and enzyme function. While Lys-450 is involved in activity that is not dependent on flavin binding, His-447 and Trp-448 are either directly or indirectly involved in interactions with flavin. After testing the *T. cruzi* GALO with alditol oxidase, Kudryashova et al. [58] postulated that His-447 and Lys-450 are situated near enough to the flavin to allow for a direct interaction. Additionally, they mentioned that His-447 might be bound by the flavin moiety. In conclusion, the flavin cofactor may be connected to the HWXK motif at the C-terminus or the FAD-binding motif at the N-terminus.

Based on the phylogenetic analysis of the AlOR sequences harboring only the HWXK motif [121] it can be concluded that the proteins lacking the HWAK motif are distantly related to GULO. It is easy to identify *GULO* genes in annotated genomes by conducting a basic Blast PHI (pattern-hit initiated) search using the HWAK motif. The HWGK motif is present in GULO encoding sequences outside of the fungi outgroup, with the exception of those from the tick, *Rhipicephalus microplus* (Canestrini, 1888) (Ixodida: Ixodidae), and the cnidarian fish parasite, *Kudoa iwatai* Egusa and Shiomitsu, 1983 (Multivalvulida: Kudoidae). The HWAK motif is present in ten *GULO*-encoding sequences from non-Bilateria and Protostomia species, which are shown as an external group to the fungal sequences in the consensus tree. According to reports, the anticipated structure of the GULO of *Mus musculus* Linnaeus, 1758 (Rodentia: Muridae) has the GULO FAD-binding domain and the HWAK motif region adjacent to one another. It is possible that this closeness is essential for creating a covalent bond with the FAD cofactor [28]. In light of these findings, it is plausible that, in contrast to the HWGK alternative, where the protein/cofactor interaction can be significantly weaker, the inclusion of an alanine at the HWAK motif confers stronger and permanent covalent binding between GULO and the relevant cofactor. This may, therefore, be connected to the different catalytic characteristics of the proteins containing the two variations of this motif.

## 8. Sequence Analysis of Rat L-gulono-γ-lactone Oxidase

The analysis of the nucleotide sequences from the human genome indicates that the *GULO* pseudogene maps to human chromosome 8p21. It accumulated numerous mutations over 40 million years ago, since it stopped being active [28,109]. At the protein level, it shows over 94% similarity to the rat GULO [80]. In terms of its amino acid identity, the vertebrate GULO shares 64–95% of its identity and has fewer conserved exons than the 12 found in the genes of mice and rats (Table 2) [122]. Remarkably, the causative agent of tuberculosis, *M. tuberculosis*, has a protein (Rv1771) encoded in its genome that shares approximately 32% of its identity with the rat GULO protein. Additionally, the *Rv1771* gene was cloned and expressed in *E. coli*, then affinity chromatography was used to purify and characterize the resulting protein [105].

## 9. Conclusions and Future Perspectives

This review shortly summarizes the role of ascorbic acid (AsA) in biological processes and discusses various AsA biosynthetic pathways, particularly in plants and animals. The main focus is on L-gulono-γ-lactone oxidase (GULO) from various species, their exact location, chemical characterization, and the kinetic parameters. We also highlight our recent results showing that the recombinant C-terminal cytoplasmic domain in rat GULO, lacking the FAD-binding site and containing only the HWAK motif, is enzymatically active when produced in *E. coli* cells. It demonstrates that the HWAK motif is sufficient for binding the flavin cofactor. This finding might shed more light on the *A. thaliana GULLO7* gene (abundantly expressed in mature pollen) and its protein product. The predicted protein is shorter than other GULLOs and aligns only with the C-terminal part of other GULOs, but might be expected to have enzymatic GULO activity, despite the absence of a FAD-binding domain.

## Figures and Tables

**Table 1:** Comparison of biochemical parameters for ALO family members characterized from different species.

Species	Molecular Mass (kDa)	Optimum pH	Optimum Temperature (°C)	K_m_ (mM)	V_max_ (µmol/min/mg Protein)	Reference
Rat liver GULO	51	7.8	37	0.066	0.63	[3]
Goat liver GULO	51	Nd	Nd	0.15	2.7	[3]
Chicken kidney GULO	50	7.3	Nd	0.007	139	[87]
Recombinant fGULO expressed in *E. coli*	50	7	40	0.053	780	[110]
Recombinant cGULO expressed in *E. coli*	20	6.5	30	0.042	374	[110]
GfGULLO	69	7	45	24	2.1	[99]
MtGLDH	70	8	40	5.5	0.041	[105]
AtGalDH	60	8.8	25	13.1	Nd	[59]
MsGULLO	70	Nd	Nd	Nd	Nd	[13]
Recombinant (AtGULLO5) expressed in *N. benthamiana*	65	9	40	33.8	4.5 × 10^−3^	[43]

Nd, not determined; fGULO, rat full-length L-gulono-γ-lactone oxidase; cGULO, rat C-terminal L-gulono-γ-lactone oxidase; GfGULLO, *Grifola frondosa* L-gulono-γ-lactone oxidase; MtGLDH, *Mycobacterium tuberculosis* L-gulono-γ-lactone dehydrogenase; AtGalDH, *Arabidopsis thaliana* L-galactono-γ-lactone dehydrogenase; MsGULLO, *Mucuna sempervirens* L-gulono-γ-lactone oxidase; AtGULLO5, *Arabidopsis thaliana* homologue of GULLO.

**Table 2:** Conservation of genomic structure of *L-gulono-γ-lactone oxidase* gene in selected mammals.

Exon Number	I	II	III	IV	V	VI	VII	VIII	IX	X	XI	XII
**Rat**	•	•	•	•	•	•	•	•	•	•	•	•
**Mouse**	•	•	•	•	•	•	•	•	•	•		•
**Guinea pig**		•	•	•		•	•	•	•	•	•	•
**Human**								•	•	•		•

The conserved exon structure of mice and rats is shown aligned with the guinea pig and human genes. Missing dots indicate the lack of a corresponding exon.

## Abbreviations

AsA, L-ascorbic acid; NO, nitric oxide; 2-KLG, 2-keto-L-gulonic acid; AlORs, aldonolactone oxidoreductases; ALO, D-arabinono-γ-lactone oxidase; FAD, flavin adenine dinucleotide; ROS, reactive oxygen species; VAO, vanillyl alcohol oxidase; GUL, L-gulono-γ-lactone; GULO, animal L-gulono-γ-lactone oxidase; GULLO, plant L-gulono-γ-lactone oxidase; GLDH, L-gulono-γ-lactone dehydrogenase; GAL, L-galactono-γ-lactone; GalDH, L-galactono-γ-lactone dehydrogenase; GALO, L-galactono-γ-lactone oxidase; Cyt c, cytochrome c.
